# Sentinel lymph node detection in thyroid carcinoma using [^68^Ga]Ga-tilmanocept PET/CT: a proof-of-concept study

**DOI:** 10.1007/s00259-023-06449-0

**Published:** 2023-09-29

**Authors:** Lisa H. de Vries, Lutske Lodewijk, Tessa Ververs, Alex J. Poot, Rob van Rooij, Lodewijk A. A. Brosens, Ronald R. de Krijger, Inne H. M. Borel Rinkes, Menno R. Vriens, Bart de Keizer

**Affiliations:** 1 Department of Surgery, University Medical Center Utrecht, Heidelberglaan 100, 3584 CX Utrecht, The Netherlands; 2 Department of Pharmacy, University Medical Center Utrecht, Heidelberglaan 100, 3584 CX Utrecht, The Netherlands; 3 Department of Nuclear Medicine and Radiology, University Medical Center Utrecht, Heidelberglaan 100, 3584 CX Utrecht, The Netherlands; 4 Department of Pathology, University Medical Center Utrecht, Heidelberglaan 100, 3584 CX Utrecht, The Netherlands; 5 Department of Pathology, Princess Máxima Center for Pediatric Oncology, Heidelberglaan 25, 3584 CS Utrecht, The Netherlands

**Keywords:** Sentinel lymph node, Thyroid carcinoma, ^68^Ga-tilmanocept PET/CT, ICG-^99m^Tc-nanocolloid

## Abstract

**Purpose:**

Sentinel lymph node (SLN) biopsy is rarely used for thyroid carcinoma staging. This is due to challenges associated with conventional Tc-99m-labeled tracers, often producing a large hotspot at the injection site, potentially hiding nearby SLNs (shine-through effect). The aim of this study was to demonstrate the feasibility and effectiveness of SLN visualization using the new PET tracer [^68^Ga]Ga-tilmanocept.

**Methods:**

Patients with thyroid carcinoma underwent ultrasound-guided peritumoral injection of [^68^Ga]Ga-tilmanocept and ICG-[^99m^Tc]Tc-nanocolloid. [^68^Ga]Ga-tilmanocept PET/CT scans were conducted at 15 min and 60 min post-injection to visualize the SLNs. SLN biopsy was performed using ICG-[^99m^Tc]TC-nanocolloid for intraoperative identification. The corresponding lymph node level was resected for reference.

**Results:**

Seven differentiated thyroid carcinoma (DTC) and 3 medullary thyroid carcinoma (MTC) patients were included, of which 6 were clinically node-negative. The median number of SLNs detected on [^68^Ga]Ga-tilmanocept PET/CT and resected was 3 (range 1–4) and 3 (range 1–5), respectively. Eight SLNs were found on PET/CT in the central compartment and 19 in the lateral compartment. The SLN procedure detected (micro)metastases in all patients except one. Seventeen of 27 pathologically assessed SLNs were positive, 8 negative, and 2 did not contain lymph node tissue, which led to upstaging in 5 out of 6 clinically node-negative patients.

**Conclusions:**

[^68^Ga]Ga-tilmanocept PET/CT identified SLNs in all patients, mainly in the lateral neck. The SLNs were successfully surgically detected and resected using ICG-[^99m^Tc]Tc-nanocolloid. This technique has the potential to improve neck staging, enabling more personalized treatment of thyroid cancer according to the lymph node status.

**Trial registration:**

2021–002470-42 (EudraCT).

## Introduction

The use of sentinel lymph node (SLN) biopsy as a diagnostic staging procedure is widespread across various cancers but is not routinely applied to stage thyroid carcinoma. The main objective of the procedure is to identify the first draining lymph node(s) from the primary tumor, referred to as SLN(s), which are most likely to contain metastases if present. A positive SLN justifies further lymph node dissection or additional treatment, while a negative SLN indicates the absence of metastasized disease, requiring less extensive treatment [1]. Several techniques are available to perform the SLN procedure, involving preoperative identification through imaging modalities such as lymphoscintigraphy, SPECT/CT, and PET/CT. Intraoperative identification of SLNs can be achieved using a collimated gamma probe in conjunction with a radiotracer or visually with the aid of dye or fluorescence. Often, multiple techniques are combined for optimal results.

The close proximity of lymph nodes to the primary tumor in thyroid cancer poses a challenge for identifying SLNs. The use of peritumoral injected [^99m^Tc]Tc-labeled radiotracers can result in a large hotspot on conventional gamma camera or SPECT imaging, potentially overshadowing smaller hotspots originating from the SLNs. This “shine-through” or “overshine” phenomenon can lead to missed SLNs and false procedures [2]. By limiting the shine-through phenomenon, the false-negative rate associated with SLN biopsy might be reduced, leading to better oncological outcomes [3]. Using a PET tracer could overcome the disadvantages of conventional Tc-99 m-based tracers as PET/CT provides dynamic 3-dimensional information with much higher spatial resolution compared to conventional gamma camera or SPECT imaging. Also, because of the better spatial resolution, the anatomic localization of SLNs is improved which is crucial in the complex neck anatomy with its abundant lymph nodes.

Differentiated thyroid carcinoma (DTC) generally has a favorable prognosis with 10-year survival rates exceeding 90% [4]. However, structural recurrent disease, primarily involving cervical lymph node metastases which could not be detected initially, can occur in up to 40% of patients. The surgical management of DTC involves hemithyreoidectomy or total thyroidectomy, depending on tumor size and stage, accompanied by lymph node dissection in case of metastases [5]. The use of SLN biopsy has been limited in DTC due to the standard treatment with radioactive iodine (RAI) to treat remaining (micro)metastases [5, 6]. In the last years, there has been a growing trend toward minimizing RAI due to its side effects. Studies have shown that low-dose RAI is as effective as high-dose RAI for low-risk patients, and recent research has demonstrated comparable results in disease-free survival between patients who did and did not receive RAI [7–10]. The SLN procedure has been investigated in DTC using various techniques, but has not been considered relevant due to the former standard treatment with RAI [11–17]. By reducing RAI administration, the chances of undiagnosed (micro)metastases receiving inadequate treatment rise. Considering this, SLN biopsy could serve as a valuable tool for selecting patients who would benefit from RAI ablation and those for whom RAI can be omitted. Additionally, since low-risk DTC patients are increasingly treated with hemithyroidectomy alone, a positive SLN could indicate the need to proceed with total thyroidectomy followed by RAI.

Medullary thyroid carcinoma (MTC) differs from other thyroid cancers as it originates from the parafollicular C-cells. Besides a sporadic form, in 25–30% of patients, MTC develops as part of the hereditary tumor syndrome Multiple Endocrine Neoplasia type 2 (MEN2) [18]. While MTC accounts for only 4% of thyroid carcinomas, it is responsible for 13% of thyroid cancer-related deaths [19]. Ten-year survival rates are 100% for stage I, 93% for stage II, 71% for stage III, and 21% for stage IV [20]. Surgery is the cornerstone of MTC treatment, while adjuvant therapy is of limited value. RAI is ineffective due to the lack of iodine uptake by MTC cells, contrary to DTC cells [21]. Treatment of clinically node-negative disease involves a total thyroidectomy and central neck dissection. This treatment strategy is based on the hypothesis that most thyroid tumors metastasize to the central compartment first before spreading any further [20]. However, approximately 61% of patients do not exhibit lymph node metastases after central neck dissection, suggesting that unnecessary lymph node dissection was performed, risking unnecessary complications [22]. Furthermore, levels of the tumor marker calcitonin remain elevated in most patients after intended curative resection, indicating the presence of subclinical active tumor tissue [23, 24]. SLN biopsy, which has only been described in MTC patients in a few papers, could select patients for more personalized treatment [25–27]. A positive SLN in the lateral compartment could identify patients who would benefit from additional lateral neck lymph node dissection, potentially leading to curation. So, the SLN procedure might not only improve the staging of thyroid carcinoma but also help to achieve curation as well as prevent unnecessary complications and reoperations.

To combine preoperative PET/CT imaging with intraoperative detection using a ^99m^Tc-tracer and a gamma probe, a PET tracer with a short half-life is necessary. 68-Gallium (^68^ Ga) possesses a half-life of 68 min and has successfully been labeled to tilmanocept [28]. Tilmanocept, which has been designed for SLN mapping, exhibits characteristics that align specifically with the requirement of the procedure. Successful SLN identification using ^68^ Ga-labeled tilmanocept imaging has been demonstrated in animal models. Furthermore, [^68^Ga]Ga-tilmanocept PET/CT has been used in patients previously at our institute [28–30].

This paper presents the results of a proof-of-concept study investigating SLN biopsy in DTC and MTC using [^68^Ga]Ga-tilmanocept PET/CT and ICG-[^99m^Tc]Tc-nanocolloid.

## Materials and methods

### Eligibility criteria

Adult patients with a cytologic diagnosis of DTC (Bethesda 6 or proven metastasis) or MTC (Bethesda 6, proven metastasis or MEN2 with elevated calcitonin) who would undergo an open hemithyroidectomy or total thyroidectomy with or without neck dissection were included.

### Study procedures

**Fig. 1:**
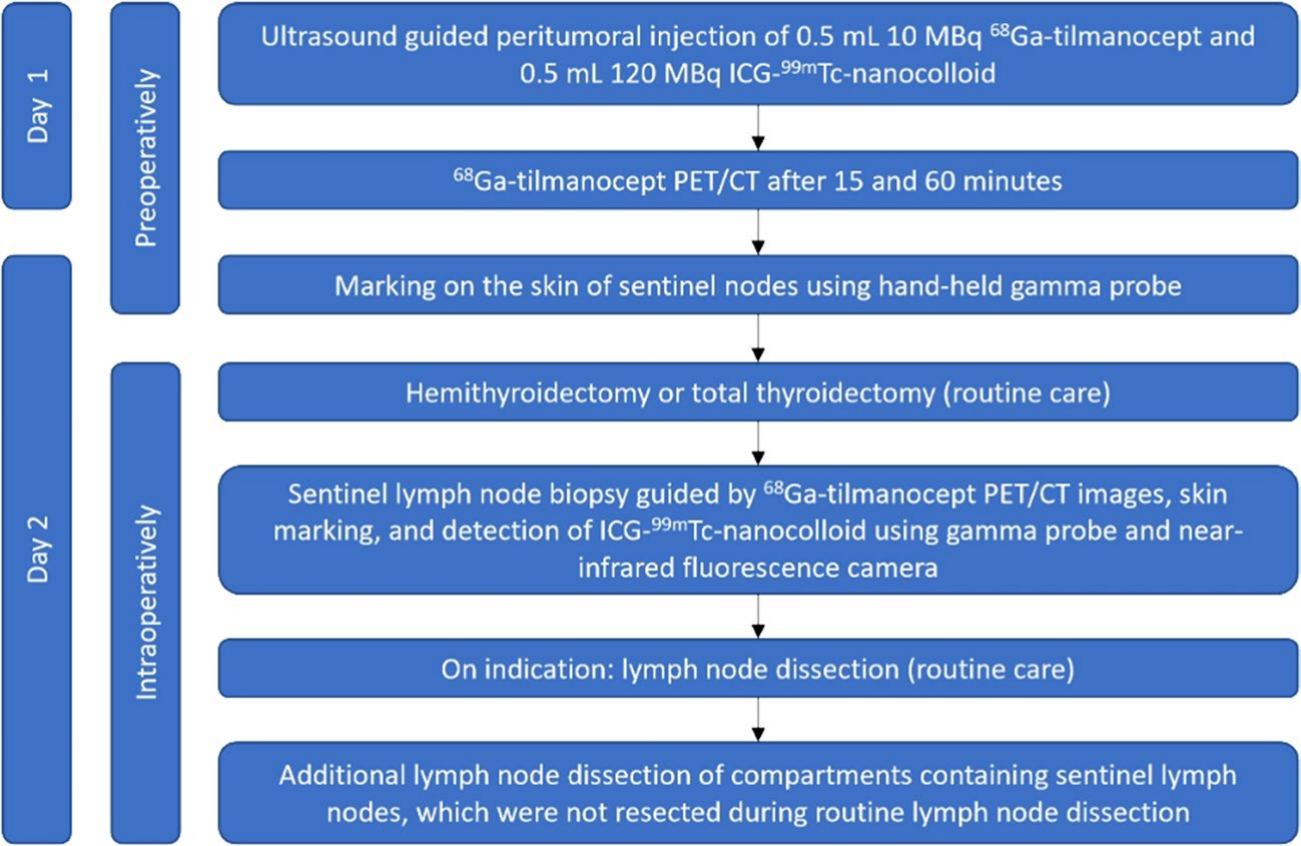
Flowchart of study procedures

Subsequently, 0.5 mL (10 MBq) [^68^Ga]Ga-tilmanocept and 0.5 mL (120 MBq) ICG-[^99m^Tc]Tc-nanocolloid were injected peritumorally using ultrasound guidance. Hereafter, patients underwent [^68^Ga]Ga-tilmanocept PET/CT scans at 15 min and 60 min post-injection, each with a 5-min PET acquisition time. Lymph nodes with activity in close proximity to the tumor and lymph nodes with high activity were considered SLNs. With knowledge of the localization seen on [^68^Ga]Ga-tilmanocept PET/CT, the approximate location of the SLNs was preoperatively assessed using a hand-held gamma camera (Crystal Cam, Crystal Photonics GmbH, Berlin, Germany) and marked on the skin [31]. During regular surgery (hemithyroidectomy or total thyroidectomy, with or without neck dissection), the SLNs were surgically removed with the help of the PET/CT images, skin markings, hand-held gamma probe and fluorescence camera. If SLNs appeared in a lymph node compartment, which was not suspected of containing lymph node metastases before entering this study, additional lymph node dissection of the corresponding level was performed. A detailed flowchart of the performed study procedures is shown in Fig. 1.

### Outcome measures/endpoints

To determine the feasibility of the SLN procedure in thyroid carcinoma, the number of SLNs determined on [^68^Ga]Ga-tilmanocept PET/CT and the number of resected SLNs were considered the primary endpoints. Secondary endpoints consist of the localization of the SLNs, the pathology result of SLNs compared to the pathology result of the rest of the corresponding lymph node levels, and the best time to perform imaging. The optimal scan time was determined by comparing the two [^68^Ga]Ga-tilmanocept PET/CTs to assess which scan is most valuable; the PET/CT performed after 15 min, after 60 min, or whether both scans were of added value in identifying the SLNs. Lymph nodes were considered second echelon nodes in the following cases: lymph nodes distal from sentinel lymph nodes with low activity. Other secondary endpoints were the complexity, feasibility, and additional value of the various identification methods used during the SLN biopsy, based on a questionnaire directed to the surgeons.

### Pathology

SLNs were processed according to the following protocol. SLNs < 5 mm were fully embedded, 5–10 mm SLNs were halved along the longest axis, and SLNs of > 1 cm were lamellated perpendicular to the longest axis. Blocks were cut at five levels with 250-µm spacing in between. All sections were stained with H&E. If no tumor cells were recognized with H&E, immunohistochemical examination was performed on all levels using cytokeratin (CKAE1/3, clone PCK26, ready to use, Roche). Antigen retrieval was performed by cooking in EDTA (CC1, Roche) for 16 min and treating with protease 3. Hereafter, the antigen was incubated for 8 min.

### Statistics

Quantitative variables were expressed as medians with ranges. A number of cases and percentages were presented for categorical variables. Missing data was not imputed. Analysis was performed using SPSS version 25.0 software (IBM, Armonk, NY, USA).

### Ethical approval

This study was approved by the Institutional Review Board of the University Medical Center Utrecht. All procedures performed in this study were in accordance with the 1964 Helsinki Declaration and its later amendments or comparable ethical standards.

## Results

### Clinicopathological characteristics

In this proof-of-concept study, 10 patients were included, of which 8 were female and 2 were male (Table 1). The median age was 54.5 years (range 27–78). Median tumor size was 26.5 mm (range 12–42 mm). Two patients had a preoperatively known/suspicious multifocal tumor. Six patients were clinically node-negative, and 4 had known lymph node metastases.

**Table 1:** Baseline characteristics

	*N* = 10
Woman	8
Median age in years (range)	54.5 (27–78)
Type	
DTC/PTC	7
MTC	3
Focality	
Unifocal	8
Multifocal	2
Clinical node status	
cN0	6
cN +	4
^68^Ga-tilmanocept PET/CT	
Median-injected dose in MBq (range)	6.50 (2.88–7.88)
Median SLNs detected per patient (range)	3 (1–5)
Total	27
Central	16
Lateral	11
15 min	
Median KBq per SLN (range)	16.3 (2.6–356.3)
60 min	
Median KBq per SLN (range)	13.8 (1.5–179.1)
Surgery	
Median SLNs resected per patient (range)	3 (1–4)
Total	27
Gamma probe, median counts per second (range)	1000 (120–3600)
Fluorescence	
Positive	24
Negative	1
Dubious	2
Pathology	
Median tumor size in mm (range)	26.5 (12–42)
SLNs	27
Positive	17
Negative	8
Other tissue	2
Pathological node status	
pN0	1
pN1a	1
pN1b	8

*DTC*, differentiated thyroid carcionma; *PTC*, papillary thyroid carcinoma; *MTC*, medullary thyroid carcinoma; *KBq*, kilobecquerel; *MBq*, megabecquerel; *SLN*, sentinel lymph node

### ^68^Ga-tilmanocept PET/CT

**Fig. 2:**
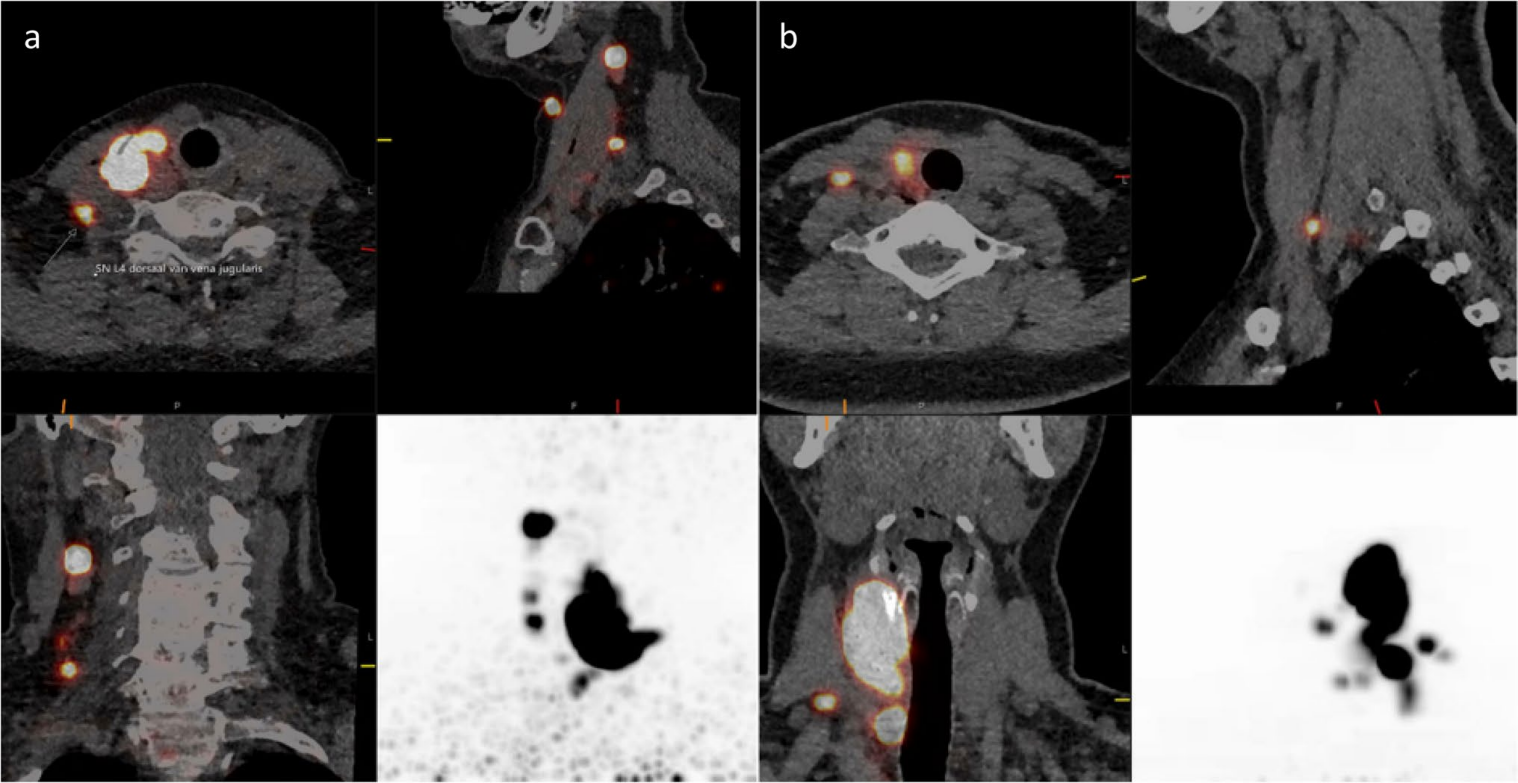
^68^Ga-tilmanocept PET/CT images of (**a**) patient no. 7 showing SLNs in levels 2 and 4 and aspecific paratracheal uptake not related to a lymph node and (**b**) patient no. 3 with SLNs in levels 3, 4, and 6

The results of the preoperative imaging, surgery, pathology, and lab are summarized in Table 2. The median-injected dose of tilmanocept was 6.50 MBq (range 2.88–7.88 MBq). The median number of detected SLNs using was 3 (range 1–5). Nineteen second echelon nodes were detected, of which 4 were newly detected on the PET/CT after 60 min. No additional SLNs were visualized on the PET/CT performed after 60 min compared to the PET/CT after 15 min. Examples of [^68^Ga]Ga-tilmanocept PET/CT imaging are shown in Fig. 2.

**Table 2:** Clinicopathological characteristics per patient

Patient	Gender	Age	Type of cancer	Size in mm	Focality	cTNM stage	pTNM stage	Preoperative calcitonin	3 months postoperative calcitonin
1	F	55	PTC	31	Unifocal	cT2N1b	pT3bN1b	NA	NA
2	F	64	PTC	46	Unifocal	cT3N0	pT3**N1b**	NA	NA
3	F	43	PTC	8	Unifocal	cT1aN1b	pT1N1b	NA	NA
4	F	78	PTC	28	Unifocal	cT3N0	pT2**N1b**	NA	NA
5	M	33	MTC	26	Unifocal	cT3N0	pT3a**N1b**	1260	< 5.0
6	F	54	MTC	47	Unifocal	cT3N0	pT3a**N1a**	11,200	20
7	F	71	MTC	23 & 25	Multifocal*	cT2N1bM1	pT2N1b	4500	780
8	F	27	PTC	9 & 29	Multifocal	cT2N0	pT2**N1b**	NA	NA
9	F	34	PTC	11	Unifocal	cT1bN1b	pT1bN1b	NA	NA
10	M	74	PTC	24	Unifocal	cT2N0	pT2N0	NA	NA

*F*, female; *M*, male; *PTC*, papillary thyroid carcinoma; *MTC*, medullary thyroid carcinoma

^*^Based on TIRADS 4 and 5

Entries in bold are upstaged due to the SLN procedure

### Surgery

Intraoperative identification was achieved using the images of the preoperatively performed [^68^Ga]Ga-tilmanocept PET/CTs, preoperatively applied skin markings, and intraoperative identification using the gamma probe and near-infrared fluorescence camera. According to the surgeons, the gamma probe was the most valuable identification tool. The median measured counts per second using the gamma probe was 1000 (range 120–3600). Twenty-four SLNs were fluorescent, one was not and two showed dubious fluorescence. The median number of resected SLNs per patient was 3 (range 1–4).

### Pathology

**Fig. 3:**
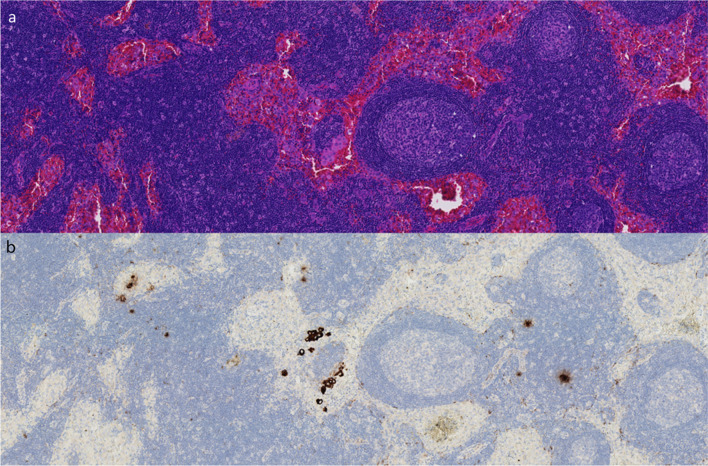
Pathology results of an SLN of a papillary thyroid carcinoma patient showing (**a**) H&E staining in which metastases are not visualized and (**b**) cytokeratin staining showing micrometastases

As shown in Table 3, 27 SLNS were resected and pathologically assessed. Seventeen SLNs were positive, of which 12 were found using H&E staining and 5 micrometastases using additional cytokeratin staining. Eight SLNs were negative. Pathological examination showed that one parathyroid and some fatty tissue were erroneously mistaken for an SLN. Examples of the H&E and cytokeratin stainings are shown in Fig. 3.

**Table 3:** Results of ^68^Ga-tilmanocept PET/CT, surgery, and pathology per patient

Patient		^68^Ga-tilmanocept					Surgery	Pathology			
	cTNM stage	Injected dose in MBq	SLNs detected	KBq per SLN after 15 min	KBq per SLN after 60 min	SENs after 15/60 min	SLNs resected	SLN	Level	Metastatic/total lymph nodes	pTNM stage
1	cT2N1b	7.24	Level 4 L Level 6 L	18.71 12.70	12.17 10.13	2/2	Level 4 L Level 6 L	Pos ®	Pos Pos	4/19	pT3bN1b
2	cT3N0	7.88	Level 3 R Level 4 R Level 4 R Level 6 R	4.24 8.90 9.92 16.34	3.36 9.68 5.27 11.23	2/0	Level 3 R Level 4 R	Pos Pos	Pos Neg	11/41	pT3N1b
3	cT1aN1b	6.50	Level 3^ L Level 4 R Level 6 R Level 6 R	2.63 14.80 229.00 13.04	2.53 17.50 153.15 11.06	3/0	Level 4 R Level 6 R Level 6 R	Pos Neg Neg	Neg Pos	5/51	pT1N1b
4	cT3N0	6.49	Level 2^ L Level 4 L Level 4 L Level 6 R	43.20 64.60 49.78 87.69	19.80 35.52 26.89 63.28	2/0	Level 4 L Level 4 L Level 6 R	Pos Pos Pos	Pos Pos	4/19	pT2N1b
5	cT3N0	6.37	Level 3 R Level 4 R Level 6 R Level 6 R	58.39 5.24 57.00 30.70	44.80 4.55 37.00 23.43	3/0	Level 3 R Level 6 R Level 6″ R Level 6″ R Level 6 R	Pos Pos Pos Neg Pos	Neg Pos	2/21	pT3aN1b
6	cT3N0	7.33	Level 4 L	26.81	17.69	0/0	Level 6”	Pos	Pos	1/16	pT3aN1a
7	cT2N1bM1	*	Level 2 R Level 4 R	10.19 3.20	6.60 2.07	1/0	Level 2 R Level 4 R Level 6 R	Pos Neg #	Pos Pos Pos	2/4	pT2N1b
8	cT2N0	*	Level 2^ L Level 4 L Level 6 L	115.54 356.28 294.03	52.76 168.87 179.12	1/0	Level 4 L Level 4 L Level 6 L	Pos Neg Pos	Neg Pos	4/26	pT2N1b
9	cT1bN1b	2.88	Level 2 L Level 4 L	4.62 9.46	6.76 13.81	1/1	Level 2 L Level 4 L	Pos Pos	Pos Pos	8/42	pT1bN1b
10	cT2N0	6.41	Level 3 L	4.49	1.45	0/1	Level 3 L Level 3 L Level 3 L	Neg Neg Neg	Neg	0/12	pT2N0

*MBq*, megabecquerel; *SLN*, sentinel lymph node; *, measurement failed; *KBq*, kilobecquerel; *L*, left; *R*, right; *SENs*, second echelon nodes; ^, preoperatively decided not to resect;”, other SLN than on PET/CT; #, parathyroid; ®, fatty tissue

### Postoperative calcitonin

Three patients with MTC were included. Patient no. 5 was treated curatively due to the resection of a metastasis in the lateral compartment which would have been missed without the SLN procedure. His calcitonin was 1260 ng/L preoperatively and decreased to < 5 ng/L after 1 month and remained unchanged after 7 months. Patient no. 6 had a preoperative calcitonin of 11,200 ng/L, which decreased to 20 ng/L after 3 months and remained stable 12 months after surgery. In patient no. 6, ^68^Ga-tilmanocept PET/CT showed an SLN in level 4 and no SLNs in level 6. During surgery, no SLNs were found in level 4 and pathology showed no metastases in this compartment. An SLN was resected in level 6 and turned out to be positive. Patient no. 7 had preoperatively known extensive metastasized disease (cT2N1bM1) with calcitonin of 4500 ng/L. Following the standard of care, this patient would have undergone a lateral lymph node dissection. However, [^68^Ga]Ga-tilmanocept PET/CT revealed a small SLN, which was located cranially in level 2, which could have been easily missed in a standard dissection. Calcitonin values decreased to 780 ng/L after 3 months and remained stable 9 months after surgery.

## Discussion

This study aimed to prove the concept of SLN biopsy using [^68^Ga]Ga-tilmanocept PET/CT in thyroid carcinoma patients. Although SLN biopsy is a standard procedure in other malignancies such as breast cancer and melanoma, it is not commonly used in thyroid carcinoma. This is the first study investigating SLN biopsy using [^68^Ga]Ga-tilmanocept PET/CT for imaging in combination with ICG-[^99m^Tc]Tc-nanocolloid for intraoperative localization. We were able to visualize SLNs with high resolution using PET/CT, followed by marking on the skin using a hand-held gamma camera and a day later the surgical removal of the SLNs using a gamma probe and fluorescence camera.

Tilmanocept is readily available and can be easily labeled with ^68^Ga. The main advantage of [^68^Ga]Ga-tilmanocept PET/CT is the short acquisition time and high resolution of PET compared to SPECT which limits the shine-through effect. Therefore, preoperative imaging using PET/CT is especially of added value to SLN imaging of the neck, with its abundant lymph nodes located near to the primary tumor in an area containing vital structures. [^99m^Tc]Tc-tilmanocept can also be used for intraoperative identification of SLNs instead of ICG-[^99m^Tc]Tc-nanocolloid. However, the use of ICG-[^99m^Tc]Tc-nanocolloid enables the confirmation of SLNs using a near-infrared fluorescence camera.

In our study, 6 patients with clinically no evidence of lymph node metastases (cN0 patients) were included. In 1 and 4 of these patients, SLNs containing metastases were found in the central neck compartment (pN1a) and lateral neck compartment (pN1b), respectively. So, of the 6 included cN0 patients, only one ended up being pN0. In 6 of the 7 DTC patients, one or more SLNs turned out positive for metastases. The percentage of metastasized DTC in our study (86%) is high compared to the 20–50% reported in the literature [5]. Our hypothesis was that SLN biopsy could select patients who are not eligible for de-escalation of treatment by omitting RAI or performing a hemithyroidectomy instead of total thyroidectomy. In our small pilot study, the opposite was true; we detected unsuspected lymph node metastases with the aid of the SLN procedure in most patients that had a larger chance of developing clinically relevant lymph node metastases or have biochemical evidence of persistent disease. SLN biopsy also seems promising for patients with MTC. Considering that the standard treatment of MTC is total thyroidectomy with central neck dissection, a remarkably high percentage of lateral metastases (80%) was found in our cohort. For MTC patients, adjuvant therapy is of limited value. Surgical resection is the cornerstone of treatment and aims to achieve loco-regional control and, if possible, curation [20, 32]. Patients who are biochemically cured with normal basal calcitonin levels after intended curative dissection have a 10-year survival rate of 97.7% [20, 33]. Therefore, adequate surgical resection is of major importance. However, standard prophylactic lateral neck dissection is not desired in all patients and leads to unnecessary risk of complications such as damage to the thoracic duct or accessory nerve resulting in shoulder dysfunction [20]. In our cohort, 5 patients had micrometastases which cannot be seen on preoperative imaging. This emphasizes that the SLN procedure can help select those cN0 or cN1a patients who can benefit from an additional lateral dissection during primary surgery to offer the best chance for curative resection, and those for whom a central neck dissection is sufficient. Therefore, we believe that SLN biopsy could be a guideline-changing procedure for patients with MTC.

SLN biopsy in MTC has been investigated in a few studies [25–27, 32]. Puccini et al. performed the SLN procedure using preoperative lymphoscintigraphy and intraoperative gamma probe and found micrometastases in 3 of 4 cT1N0 patients. Only the SLNs were removed without resection of the compartments, leading to biochemical curation in all patients with a mean follow-up time of 30.5 months [25]. Kim et al. used preoperative lymphoscintigraphy, a collimated gamma probe, and a frozen section to identify SLNs in 14 of 16 cN0/cN1a MTC patients. Four and three patients were found to have metastases in the central and lateral compartments, respectively [27]. Boni et al. describe radioguided SLN biopsy in a cN0 patient. Micrometastases were found in two lateral SLNs with consecutive lateral neck dissection, resulting in undetectable calcitonin values during follow-up [26]. Santrac et al. performed the SLN procedure using methylene blue dye and frozen section in 20 cN0M0 microcarcinoma patients with calcitonin values < 1000 pg/mL. Metastasized SLNs and additional metastases were found in the lateral compartment of 2 patients [33].

In our study, 5 of the 17 positive SLNs contained micrometastases, which were detected using additional cytokeratin staining. The use of a frozen section does not seem suitable for SLN biopsy in thyroid carcinoma since these micrometastases would have been missed. By omitting the frozen section, resection of the whole level in which the SLN is located during primary seems indicated to avoid resurgery in previously operated tissue. On the other hand, Puccini et al., as described earlier, performed curative resection by only resecting the SLN and without performing lateral neck dissection [25]. In order to minimize the need for reoperations in previously resected areas, it seems advisable to maintain the standard central neck dissection, even in cases where no SLNs are identified in the central compartment. The extent of dissection should be further assessed in a larger group of patients.

The limitations of this study are mostly due to the nature of a proof-of-concept study. Only a few patients were included. Also, clinically node-positive patients were included, which limits the interpretation of clinical relevance, although this was not a primary endpoint in this study.

## Conclusions

SLN procedure using [^68^Ga]Ga-tilmanocept PET/CT combined with ICG-[^99m^Tc]Tc-nanocolloid was able to detect and resect SLNs in all patients. A remarkable number of lateral lymph node metastases was found in our cohort. Introduction of the SLN procedure in thyroid carcinoma may lead to more accurate staging, prevent unnecessary treatment and accompanying complications, and select patients in need of more extensive treatment, enabling (earlier) curation.

## Data Availability

The datasets used and/or analyzed during the current study are available from the corresponding author upon reasonable request.
